# Structure and legibility of informed consent for clinical practice in Ecuador

**DOI:** 10.3389/fmedt.2026.1774341

**Published:** 2026-03-11

**Authors:** Cristina Elisabeth Urgilés-Barahona, Xavier Astudillo-Romero, Gloria Carrión, Diego Gómez-Correa, Weneper Rojas, Alfonsina Benavides

**Affiliations:** Faculty of Health Sciences, Universidad Técnica Particular de Loja, Loja, Ecuador

**Keywords:** health, hospital, informed consent, language, legibility, noncompliance, regulatory compliance

## Abstract

**Introduction:**

Informed consent is an essential process in healthcare. It is a right endorsed by the Universal Declaration of Bioethics and Human Rights and allows people to make free and informed decisions about their health. The structure of the informed consent forms is evaluated in accordance with the regulations established in the Management Model for the Application of Informed Consent in Health Care in Ecuador.

**Methods:**

A descriptive cross-sectional study was conducted. The assessment instruments were developed based on current national regulations. A total of 482 informed consent forms from 62 health institutions in the country were evaluated.

**Results:**

Compliance with the structure established by national regulations is heterogeneous, ranging from 74.46% to 94.87%. Although these values suggest a relatively high level of adherence, they are insufficient to ensure fully informed decision-making that guarantees both patient autonomy and safety. Readability was 45.23%, which made it difficult to understand properly.

**Conclusion:**

Informed consent in the Ecuadorian context presents deficiencies in compliance with its structure, which compromises its completeness, and a significant gap in its legibility, which compromises informed decision-making and limits the exercise of patient-centred medical practice.

## Introduction

Informed consent (IC) is an essential process in medical practice and constitutes the fundamental manifestation of the principle of autonomy. Supported by the Universal Declaration on Bioethics and Human Rights, the IC recognizes the right and ability of individuals to exercise control over decisions inherent to their care and treatment, in a free and conscious manner, stressing that this right must be based on the values and beliefs of the person, as well as on adequate information. it can be removed at any time without entailing any disadvantage or detriment to the person involved (1–3). This ability to consent can be affected by factors such as discrimination, poverty, lack of access to resources and services, or specific life conditions (elderly, children, pregnant women, adolescents, chronically or terminally ill), which place the person in a state of vulnerability, and face a greater risk of harm, so they are considered vulnerable and require a different approach in the process of applying informed consent (4–6). In the context of health care, an IC that prioritizes the information, understanding, and self-determination of the person in relation to their health care can reduce vulnerability.

Another aspect to consider is the degree of understanding of the subject about the information existing in the IC. Some people (such as illiterate people or those with low academic education) have basic reading skills, recognize simple terms or relate them only to everyday knowledge and although they can identify the main theme of the text; it is difficult for them to learn from it and use its information as a tool to decide, which constitutes a limitation for their understanding and determination (7). Seen in this way, the IC must guarantee readability by maintaining a friendly structure with content adapted to the characteristics of the target population. Your text must maintain typographic and linguistic characteristics that allow it to be read and understood easily, due to its clarity, structure, vocabulary and the complexity of the language used (8, 9). In other words, legibility can be considered both typographic and linguistic, with typography allowing visual perception in relation to the form of the text and linguistics allowing the understanding of the message (9, 10). It is therefore essential that the IC is written in clear and simple language, avoiding technical terms or jargon that may be confusing for some people. Information should be presented in an organized and coherent manner, using a readable format that facilitates understanding (11). However, IC readability is influenced by inadequate writing, without considering the proper application of typography, grammar, and vocabulary.

An IC with poor readability ineffectively transmits information to the person, which can lead to ethical and legal implications. Providing from expert to layperson, detailed information about the proposed intervention, treatment or procedure, including risks, benefits and alternatives, with wording adapted to your instruction and understanding capacity, will facilitate the understanding of the procedure and ensure an informed decision without external pressure (12, 13). The expert-layman relationship demands, therefore, an adaptation by the expert of the information that will be transmitted to lay people to ensure their understanding and decision (13). For Morales Valdivia (14), the freedom and awareness of choice cannot exist without a proper understanding of the information.

In medical practice, obtaining the IC prior to clinical or surgical interventions and treatments is a legal requirement that is granted, freely and voluntarily, by the person to support their autonomy. It is made up of a written part in which the legal implications of the document are expressed and an oral part in which the doctor informs the patient what is part of this process (15). Currently, 18 Latin American countries have specific regulations for IC that describe its verbal or written application, but each country details specifications of use and application situations (11). The characteristics of structure and content of the IC are determined in the different regulations, however, there are general parameters such as: including clear, brief information, in simple language and that is appropriate to the level of education of the person.

In Ecuador, the IC is a requirement and a right guaranteed in the Constitution of the Republic in its Art. 362 (16), and the Organic Law on Health in its Art. 7 (17). The Code of Medical Ethics of Ecuador, in its articles 15 and 16, indicates the obligatory nature of IC in relation to clinical or surgical interventions and treatments (18) at each of its levels and institutions of both the Public Network and the Complementary Health Care Network. This provision is ratified in Ministerial Agreement 5316 (19) and guarantees that individuals are duly informed about the risks, benefits and procedures involved, respecting their dignity, autonomy, privacy and intimacy.

However, despite the progress made in the application of IC, and the different laws and regulations that promote its implementation, the structure and readability of IC information still do not allow for its proper application. According to data from the National Institute of Educational Evaluation, the reading comprehension performance of 70% adults is between level 1 and 2 in a category of 1–5 (20). This is where the importance of informing people accurately and clearly is born, seeking to understand the information exposed. The objective of this research was to evaluate the structure of ICs in accordance with the regulations established in the Management Model for the Application of Informed Consent in health care, based on the readability of the content, allowing the identification of opportunities for improvement to guarantee the right to information and the autonomy of each patient.

## Methodology

### Design and planning

A study was carried out with a quantitative, descriptive and cross-sectional approach. Data was obtained on the structure and readability of informed consent, without involving patients’ personal data. The project was approved by the Human Research Ethics Committee of the University of Cuenca (Ecuador), with approval code: 2023-007EO-VIUC.

### Population and sample

The institutions belonging to the Comprehensive Public Health Network (CPHN), located in different provinces of Ecuador, made up the study population. Convenience sampling was applied whose unit of analysis was each institution that agreed to participate in this research. 85 institutions belonging to the Public and Comprehensive Health Network of Ecuador were invited, selected under criteria of geographical accessibility and availability of resources. Of these, 62 institutions agreed to participate, allowing the structure and legibility of IC forms to be evaluated in procedures that require their mandatory application. Data collection was carried out between March and August 2023.

### Data collection instrument

A form was created that made it possible to characterize the institutions, in accordance with the provisions of the Regulations to establish the typology of health establishments of the National Health System, approved in 2020 by Ministerial Agreement No. 00030-2020 and in force at the time of the study (21).

To evaluate the structure of the IC, a checklist was designed based on the requirements established in the Management Model for the Application of Informed Consent in Healthcare Practice –Ministerial Agreement 5316, 2016– (22). This regulation is mandatory in the institutions of the Comprehensive Public Health Network. The resulting instrument consists of 25 dichotomous items (compliance/non-compliance) divided into seven sections, rigorously aligned with the legal standards in force in Ecuador. Each category was evaluated on a scale of 100%, assigning the items a uniform weighted value according to the number of items that make up each section. Overall compliance was obtained by averaging the scores obtained in all categories.

Legibility was measured using an instrument composed of 16 dichotomous questions (yes/no), organized in two dimensions: linguistic (4 items) and typographic (12 items). The content validity of content was evaluated by a panel of six experts, achieving a validity index of 0.98 with the application of the Lawshe Content Validity Index. Reliability was confirmed by internal consistency analysis, reporting a KR20 = 0.80.

### Results

Data from 482 informed consents from 62 health institutions in different cities of Ecuador were processed. 78.80% of the institutions included belong to the Public Health Care Network; 63.6% were included in the second level of care and 54.8% of the ICs analyzed were specific (Table 1).

**Table 1:** Characterization of participating institutions (*N* = 482).

Domain	*N*	Percentage	CI 95%	
			Lower	Upper
Type of provider				
Public network	380	78.80	75.00	82.30
Complementary network	102	21.20	17.70	25.00
Level of complexity				
Level I	61	12.70	9.90	15.80
Level II	307	63.60	59.30	67.90
Level III	114	23.70	20.00	27.60
Informed consent form				
Specific	264	54.80	9.90	15.80
Generic	196	40.70	59.30	67.90
No specific	22	4.60	20.00	27.60

When evaluating the construction of informed consent by dimensions of structure and quality of information, it is observed that, in the structure, there is less compliance with the data required for the refusal of informed consent (74.94%); the criterion “*list all complications”* showed the lowest compliance (52.3%). The second dimension with low compliance is the description of post-procedure management, which includes patient responsibilities with 78.8% compliance. In the dimension “*description of the procedure”,* the criterion with the lowest compliance (74.5%) was the presence of a graph previously selected for the form (Table 2). Reflecting that ICs have a wide gap between compliance with the standard and the reality of care. This gap could lead to failures in patient safety and lack of legal protection for the professional.

**Table 2:** Structure of informed consent.

Criteria	*N*	%
General data		
Title of the form, according to the medical IC procedure	439	91.10
Header	436	90.50
Fecha y hora	439	91.10
ID number	471	97.70
Patient's full names	466	96.70
Type of care: inpatient or outpatient	423	87.80
Name of primary diagnosis	408	84.60
Compliance	Mean: 91.34 DE: 17.44	
Description of the procedure		
Name of the procedure and type of therapeutic diagnosis	436	90.50
What does it consist of?	439	91.10
How is it done?	427	88.60
Approximate duration	403	83.60
Previously selected chart for the form	359	74.50
Compliance	mean: 85.64 DE: 28.21	
Benefits and risks		
What is it for?	375	77.80
Brief and simple explanation of the objective of the intervention and benefits	409	84.90
Risks What risks can there be?	417	86.50
Common (serious) risks	417	86.50
Rare (serious) risks	417	86.50
Specific patient-related risks	388	80.50
Compliance	mean: 83.61 DE: 27.29	
Alternatives to the procedure		
Information about alternatives to intervention	398	82.57
Compliance	mean: 82.57 DE: 37.97	
Post-procedure management		
Description of aftercare (includes patient responsibilities)	380	78.80
Compliance	mean: 78.80 DE:40.88	
Declaration of informed consent		
Declaration of informed consent^a^	457	94.80
Compliance	mean: 94.87 DE: 22.19	
Refusal and revocation of informed consent		
Foreseeable consequences if the procedure is not carried out	403	83.60
List of all complications	252	52.30
Refusal of consent data	404	83.80
Consent revocation data	396	82.20
Compliance	mean: 75.46 DE: 32.29	

a Structure in accordance with Ministerial Agreement 5316-Informed Consent, pp. 55.

The legibility of informed consent was evaluated with linguistic and typographic evaluation criteria, finding that the linguistic dimension has a percentage of compliance of 45.23%, the criterion with almost zero compliance (1.20%) was the *existence of ICs adapted to the language understood by the patient,* followed by *the existence of a process to have available readers or translators* with 10.6%. On the other hand, the typographic dimension had a compliance of 74.49%; The criterion with the lowest compliance was *verifying readability* with 38% followed by the *use of personal pronouns* (53.7%) (Table 3). Given these findings, it could be mentioned that IC ceases to be a tool for patient protection, but becomes a care requirement that must be fulfilled. The patient could often give blind consent due to the trust they place in the health personnel. Likewise, insufficient understanding of the content of the IC could lead to the patient not understanding some of the phases of their treatment, which can trigger a nocebo effect and anxiety. For the health professional, it would lead to legal consequences, because an IQ must guarantee real comprehensibility and this must be in accordance with the average educational level of a population.

**Table 3:** Readability of informed consent.

Criteria	*N*	%
Linguistic dimension		
Are there informed consents adapted to the language that the patient understands?	6	1.20
Is there a process for having readers or translators available?	51	10.60
Is the use of words in another language avoided?	442	91.70
Is medical jargon replaced with simple language?	373	77.40
Compliance	mean: 45.23 DE: 16.92	
Typographic dimension		
Does the form use sentences written in the third person?	292	60.60
Are there comprehensible titles in the sections?	449	93.20
Are personal pronouns used?	259	53.70
Are words with few syllables (no more than three syllables) used?	287	59.50
Is writing sentences written in the passive voice avoided in writing?	368	76.30
Is writing a double negative avoided in the newsroom?	414	85.90
Does consent prevent the use of symbols, numbers, and percentages?	415	86.10
Can the font and font size be easily read?	405	84.00
Is it spelled correctly without spelling mistakes?	413	85.70
Are there less than 1,000 words in the consent?	413	85.70
Are short sentences used?	411	85.30
Has the text been tested with a known evaluation tool to check for readability?	183	38.00
Compliance	mean: 74.49 DE: 19.09	

It was also sought to identify whether the IC in its structure and readability shows significant differences between the type of provider, considering that public institutions could show greater adherence to compliance with national regulations. This approach was confirmed by significant differences (*p* < 0.05) in all sections of the IC structure, evidencing a higher degree of compliance in the Public Network compared to the Complementary Network. However, no differences in legibility were identified in its linguistic and typographic dimension. This result reflects that the Ecuadorian watchdog needs to improve the monitoring of compliance in the CPNH and that institutions must adhere to compliance.

Additionally, the presence of differences in the structure and legibility of IC between public and private providers was analyzed, under the assumption that public institutions could show greater adherence to national regulations (Table 4). The results showed statistically significant differences (*p* < 0.05) in all structural sections of the IC, with a higher degree of compliance in public schools. On the other hand, legibility, both in its linguistic and typographic dimensions, did not show significant differences between the different types of providers. These findings suggest that, beyond the type of institution, gaps persist in the quality of informed consent, which raises the need for the governing body to strengthen its control, monitoring, and technical support mechanisms to ensure a homogeneous and effective implementation of the regulations throughout the health system.

**Table 4:** Bivariate analysis.

Criteria	Type of provider			Level of complexity			
	Public network	Complementary network		Level I	Level II	Level III	
	Mean	Mean	*P*	Mean	Mean	Mean	*p*
Structure							
General data	93.31	84.03	<.001	82.43	92.74	92.35	<.001
Description of the procedure	89.58	70.98	<.001	68.19	88.33	87.71	<.001
Benefits and risks	84.14	78.18	<.001	73.77	83.44	89.32	<.001
Alternatives to the procedure	85.26	72.55	.002	62.29	83.71	90.35	<.001
Post-Procedure Management	82.10	66.66	.003	59.01	79.15	88.59	<.001
Declaration of informed consent	96.05	90.19	<.001	95.08	93.48	98.26	.147
Refusal and revocation of informed consent	77.96	66.17	.001	53.27	75.97	85.96	<.001
Readability							
Linguistics	45.19	45.34	.944	48.77	43.73	47.36	.031
Tipográfica	74.51	74.42	.967	66.39	72.55	84.06	<.001

When contrasting with the levels of complexity, significant differences (*p* < 0.05) were identified in all categories. It can be seen that the lowest degree of compliance corresponds to the institutions of the first level of care. This is probably because the procedures are considered low risk, which leads both professionals and patients not to attribute relevance to IC. Another possible explanation lies in the existence of limited resources and deficiencies in the management system, considering that the first level is not required to have Healthcare Ethics Committees, whose functions include ensuring the proper management of the IC.

## Discussion

Informed consent, as an essential component of clinical practice, materializes the principle of autonomy and establishes a care relationship based on respect for the person's decision-making capacity. Its legal and ethical value is achieved when it transcends regulatory compliance and its communicative function is fulfilled: to offer sufficient, understandable and contextualized information that allows the person to make free, informed decisions consistent with their values and needs. In this sense, several authors have pointed out that informed consent should be understood as a dynamic process, rather than as an isolated act or a standardized form, integrating clear, structured information adapted to the patient's clinical and sociocultural context (3, 4, 25). In everyday practice, the effective application of informed consent is limited by structural, organizational and communicational factors. When healthcare management models focus on regulatory compliance, or succumb to the pressure of care and the fragmentation of clinical time, they turn informed consent into an administrative requirement, weakening its informative and deliberative dimension (25–27). This tension between form and substance is consistently reflected in studies carried out in different contexts and levels of care.

In the present study, the structure of informed consent was evaluated based on Ecuadorian regulations, evidencing compliance between 74.46% and 94.87%. These findings reveal significant impairments that may limit patients’ ability to make informed decisions, similar to what was reported by Gebreanenia et al. in Ethiopia, where only 35.8% of surgical patients received six or more of the 13 essential components of informed consent (23). Similarly, Ababulgu et al. observed compliance between 72.92% and 98.88% in patients undergoing cesarean sections (26). In Pakistan, Munawar et al. reported compliance between 3% and 76.5%, when analyzing ten components of consent in emergency procedures (5). Similarly, in Italy, Valente et al. identified a quality of prepartum information that ranged from 18.6% to 87.4% (28), and in Portugal, Vieira et al. found compliance levels between 21.8% and 47% when auditing basic criteria in surgical medical records (24). Finally, García-Álvarez et al. evaluated the formal quality and compliance of informed consent forms in critical areas, finding that the forms examined frequently lacked essential elements that would guarantee adequate understanding of patients (29).

Informed consent seeks to make the patient understand the proposed procedure, its risks, benefits, and alternatives. Compliance with these parameters in the present study was 85.64% for the description of the procedure, 83.61% for risks and benefits, 82.57% for therapeutic alternatives and 78.8% for post-treatment management, accounting for a significant proportion of patients who decide with incomplete information. Similarly, Ahsan et al. in 2020, examined the effectiveness of informed consent in surgical patients and found that although many patients managed to understand the nature of the procedure and its risks, only 76% indicated that they had a full understanding of all relevant aspects. Research in other contexts points to much lower compliance. A study in Ethiopia showed that only 8.4% of patients received information about the expected duration of surgery, 45.2% about possible complications, and 15.2% about therapeutic alternatives (23). In Italy, although 87.4% of women undergoing cesarean section received information on risks and benefits, in vaginal delivery the levels were considerably lower for interventions such as episiotomy or instrumental delivery (28).

In Poland, Miszewski et al. concluded that prostate biopsy patients were often unaware of the advantages and disadvantages of different procedures and that this lack of knowledge can affect their ability to make informed decisions about their care (30). Even more critical results have been reported in Pakistan, where adherence was 67.5% for the purpose of treatment, but declined to 9.5% for therapeutic alternatives, 14.8% for risks, and 20.1% for benefits (27). In Portugal, less than a quarter of consents included adequate information about benefits, risks, or complications (24). In the United States, Spatz et al. showed that, although 30% of the documents mentioned the procedure, only 11% explained its execution, 2% described risks, and 17% therapeutic alternatives (31), which confirms that insufficient information is not exclusive to health systems with fewer resources. It is important, therefore, that the IC approach is patient-centered, seeking in him, the understanding and retention of information, aspects that must be carefully weighed when evaluating the effectiveness of informed consent in different medical settings.

Refusal of a medical procedure is an intrinsic aspect of informed consent. Any person can refuse any treatment or procedure, even if it is necessary to improve their health, however, the consequences of refusing a procedure are often not discussed in detail with the patient. In this sense, the present study showed an overall compliance of 75.46% and within this category, information about all complications is included in 52.3% of ICs and information on the foreseeable consequences of refusing IC exists in 83.6% of cases. This gap is consistent with what was found in Ethiopia, where only 57.7% of patients were informed about the consequences of rejecting the proposed intervention (23), and in Portugal only 21.8% of the medical records included information on the risks of not accepting the treatment or validated alternatives (24). These findings suggest that refusal of consent continues to be treated as a marginal event, despite its ethical and legal relevance.

In South America, evidence on the structure and quality of informed consent is still limited. In Peru, low levels of compliance have been identified in the description of the duration of the procedure and its contraindications, with intermediate performance in aspects such as prognosis, therapeutic alternatives, and refusal of consent, which shows persistent structural deficiencies. In Brazil, although the majority of consents mention the type of procedure, only a minority describe it technically, which reinforces the idea of a formally complied but informationally weak consent (32).

Readability and understanding of informed consent are critical aspects of ensuring that patients can make informed decisions about their health care. Legibility further reduces power asymmetries and prevents subtle forms of structural coercion. In this study, readability reached 45.23%, a value comparable to that reported in Australia, where obstetric forms presented levels between 37.7% and 51.4% (33). In Spain, studies on anesthetic consent have shown indices that correspond to moderately difficult texts. More than half of the patients did not fully understand the document (34, 35). Something similar was evidenced in Belgium, where most of the consents evaluated presented complex reading levels (36). A systematic review conducted by García-Álvarez and García-Sánchez concluded that most informed consents written in Spanish present reading difficulties, with a high proportion classified as “somewhat difficult” or “difficult”, regardless of the index used (37).

Finally, linguistic adaptation constitutes a critical aspect of informed consent. In this study, only 1.2% of the forms were adapted to the patient's language. This figure is markedly lower than that reported by Molina et al. in Boston, where, even with forms in Spanish, Spanish-speaking women had less understanding of obstetric consent compared to English-speaking women, especially in the identification of risks such as cesarean section (38). These findings highlight that the absence of linguistic and cultural adaptation not only limits understanding, but also deepens structural inequities. In a plurinational and multicultural country like Ecuador, this lack acquires particular ethical, clinical and health relevance.

## Conclusions

Informed consent (IC) in the Ecuadorian context presents a moderately acceptable structural compliance. Although compliance with the 26 required sections ranges from 74.46% to 94.87%, this range allows many patients to decide without important information because it implies that between a quarter and a tenth of the mandatory data is missing from many forms.

Important aspects such as post-procedure management, IC refusal-revocation, and the complete list of complications describe significant percentages of non-compliance. While 8 out of 10 ICs indicate what the patient should do after the intervention, 7 out of 10 explain the steps to deny or revoke the IC, only 1 out of 2 details all the possible complications.

Linguistic readability in general is poor and culturally insensitive. Only 45.23% of the language criteria are met and only 1.2% of the forms are adapted to the patient's language or literacy level. This lack of accessibility, coupled with the limited availability of interpreters (10.6%), increases vulnerability and can ethically invalidate the IQ process.

The quality of the IC depends significantly on the type of provider and the level of hospital complexity. The institutions of the Public Network outperform the Complementary Network, for example, in the description of the procedure (89.6% vs 71.0%) and in general data (93.3% vs 84.0%). Similarly, level III hospitals show better overall scores than those at level I (*p* < 0.001), which suggests the influence of more robust resources and quality systems.

## Data Availability

The dataset generated in this research is not publicly available due to ethical and legal restrictions. However, it can be requested from the authors upon prior request.
